# Predictive factors of occult neck metastasis in patients with oral squamous cell carcinoma^☆^

**DOI:** 10.1016/j.bjorl.2015.09.005

**Published:** 2015-12-17

**Authors:** Renato Fortes Bittar, Homero Penha Ferraro, Marcelo Haddad Ribas, Carlos Neutzling Lehn

**Affiliations:** Hospital do Servidor Público Estadual (IAMSPE), São Paulo, SP, Brazil

**Keywords:** Head and neck neoplasms, Oral neoplasms, Survival outcomes, Lymph node metastasis, Prognosis, Squamous cell carcinoma, Neoplasias de cabeça e pescoço, Neoplasias orais, Resultados de sobrevida, Metástase linfática, Prognóstico, Carcinoma espinocelular

## Abstract

**Introduction:**

It is well established that cervical lymph node metastasis is the most important prognostic factor in patients with oral squamous cell carcinoma of the upper aerodigestive tract. The definition of parameters and classifications that could separate patients in groups of low, intermediate and high-risk is being attempted for several years.

**Objective:**

The objective of this study was to determine possible predictive factors related to the occurrence of occult cervical lymph node metastasis through the analysis of histopathological reports of surgical specimens obtained after oral squamous cell carcinoma resection and selective neck dissections of patients initially classified as N0.

**Methods:**

This was a primary, retrospective, observational, case–control study. Histopathological reports were reviewed to determine if some findings were related to the occurrence of occult lymph node metastasis. The events analyzed were oral cavity subsites, pT-stage, muscular infiltration, desmoplasia, vascular emboli, perineural infiltration, tumor thickness and compromised margins.

**Results:**

Occult cervical metastasis accounted for 19.10 percent of the cases. Desmoplasia, perineural infiltration, tumor thickness and pT4a stage are predictive factors of occult neck metastasis (*p*-value = 0.0488, 0.0326, 0.0395, 0.0488, respectively).

**Conclusion:**

The accurate definition of predictive factors of occult cervical metastasis may guide the selection of patients that should be referred to radiotherapy, avoiding the unnecessary exposure of low-risk patients to radiation and allowing a better regional control of the disease in those of moderate or high risk.

## Resumo

**Introdução:**

Já é bem estabelecido que a metástase oculta em linfonodo cervical é o fator prognóstico mais importante em pacientes com Carcinoma epidermóide de boca (CEB) do trato aerodigestivo superior. Há anos pesquisadores tentam definir parâmetros e classificações que poderiam separar os pacientes em grupos de baixo, médio e alto risco.

**Objetivo:**

O objetivo deste estudo foi determinar possíveis fatores preditivos relacionados com a ocorrência de metástase oculta em linfonodo cervical, por meio da análise de laudos histopatológicos de espécimes cirúrgicos obtidos após ressecção de CEB e dissecções seletivas do pescoço em pacientes inicialmente classificados como N0.

**Método:**

Este foi um estudo primário, retrospectivo, observacional e de caso-controle. Laudos histopatológicos foram revisados para determinar se alguns resultados estavam relacionados com a ocorrência de metástases em linfonodos oculto. Os eventos analisados foram: subsítios dentro da cavidade oral, estágio-pT, infiltração muscular, desmoplasia, embolia vascular, infiltração perineural, espessura do tumor e margens comprometidas.

**Resultados:**

Metástases cervicais ocultas foram responsáveis por 19,10% dos casos. Desmoplasia, infiltração perineural, espessura do tumor e estágio pT4a foram fatores preditivos de metástase cervical oculta (p = 0,0488, 0,0326, 0,0395, 0,0488, respectivamente).

**Conclusão:**

A definição precisa dos fatores preditivos de metástase cervical oculta pode orientar a seleção de pacientes que devem ser submetidos a radioterapia, evitando a exposição desnecessária dos pacientes de baixo risco à radiação, e melhorar o controle regional da doença em pessoas de risco moderado ou alto.

## Introduction

It is well established that cervical lymph node metastasis is the most important prognostic factor in patients with oral squamous cell carcinoma (OSCC) of the upper aerodigestive tract.^1^, ^2^, ^3^, ^4^

There is a distinct relationship between the number and level of cervical nodes compromised, capsular rupture and positive margins with five-year survival.^1^, ^2^, ^3^, ^4^, ^5^

Neck palpation during clinical exam still has great value; however, it has been shown that this may result in a false-negative rate of around 28.9 percent.^2^ Palpation sensitivity was estimated at 75 percent, and specificity at 83 percent, against 81 percent of sensitivity and 83 percent of specificity shown by computed tomography. Other authors demonstrated palpation sensitivity at 82 percent and specificity at 80 percent and found no significant differences comparing with cervical ultrasound.^6^, ^7^

Neck dissection is well accepted for defined evidence of cervical lymph node metastasis; however, there is still great controversy about the type and extension of neck treatment in clinically negative cervical disease.^2^, ^3^, ^4^, ^5^

The definition of parameters and classifications that could separate patients in groups of low, intermediate and high risk is being attempted for several years. Studies have implied factors such as histologic grade, muscular infiltration, desmoplasia, vascular emboli, perineural infiltration, tumor thickness and compromised margins.^5^, ^8^, ^9^, ^10^, ^11^

The presence of these multiple variables of recurrence was a precise indicator of poor prognosis.^5^, ^12^, ^13^ Other studies have shown that lymphatic or vascular invasion is an independent risk factor.^14^, ^15^

The objective of this study was to determine possible predictive factors related to the occurrence of occult cervical lymph node metastasis through the analysis of histopathological reports of surgical specimens obtained after OSCC resection and selective neck dissections of patients initially classified as N0.

## Methods

This was a primary, retrospective, observational, case–control study.

Data was obtained from a single institution. The research was performed from 1995 to 2014.

Histopathological data was obtained using chart numbers. No intervention was performed and no attempt to contact patients to obtain any kind of information was made. Patient's names or any other means of identification were not used. This study was in accordance with all principles of the Helsinki Declaration.

The search started with the gathering of all records involving oral cancer. After eliminating all double entries, we reviewed all patients’ charts and selected every patient that was initially classified as N0.

Exclusion criteria were double entries, incomplete chart information, patients without surgical conditions or who refused surgical treatment, patients who had the neck staging changed at the pre-operative period, patients with tumors smaller than 1 cm who were not submitted to elective neck dissection.

Patients were then distributed into two groups:

Group A: patients N0 who were submitted to oral cancer resection with elective neck dissection (levels I, II and III) and the absence of occult lymph node metastasis.
Group B: patients N0 who were submitted to oral cancer resection with elective neck dissection (levels I, II and III) and the presence of occult lymph node metastasis.

Histopathological reports were then accessed to determine if some findings were related to the occurrence of occult lymph node metastasis. The events analyzed were oral cavity subsites, pT-stage, muscular infiltration, desmoplasia, vascular emboli, perineural infiltration, tumor thickness and compromised margins. These were individually analyzed to determine whether those events could be related to occult lymph node metastasis with statistical significance, and if it could be used as predictive factor to determine the presence of node metastasis in clinically negative necks.

## Results

Initially, we retrieved 183 patients, and after the exclusion criteria remained with 157 patients. One hundred and twenty-seven patients did not present with occult lymph node metastasis and were included in group A. The remaining 30 patients were included in group B. Occult cervical metastasis accounted for 19.10 percent of the cases (30/157).

In group A, the average age was 65.4 years (47–88 years old). Males were 104, and females 23. In group B, the average age was 63.7 years (44–91 years old). Males were 26, and females 4.

The compromised oral cavity subsites were tongue 54.14 percent (68 cases in group A and 17 in group B), floor 29.93 percent (39 cases in group A and 8 in group B), retromolar area 11.46 percent (15 cases in group A and 3 in group B), and buccal mucosa/lips with extension to buccal mucosa 4.45 percent (5 cases in group A and 2 in group B). All of the cases in the gingiva were smaller than 1 cm and submitted to local resection with safety margins without elective neck dissection – therefore, were not included (Table 1).

**Table 1:** Demographics and occurrence subsites.

	Group A	Group B
*Gender*		
Male/female	104/23	26/4

*Age*		
Average (range)	64.4 (47–88)	63.7 (44–91)

*Oral cavity subsite*		
Tongue	68	17
Floor	39	8
Retromolar area	15	3
Buccal mucosa/lips with extension to buccal mucosa	5	2

Odds ratio, confidence interval and *p*-value according to oral cavity subsites are shown in Table 2.

**Table 2:** Odds ratio, confidence interval and *p*-value according to oral cavity subsites.

	OR	95% CI	*p*-Value
Tongue	1.1346	0.5088–2.5300	0.7576
Floor	0.8205	0.3360–2.0035	0.6641
Retromolar area	0.8296	0.2241–3.0714	0.7797
Buccal mucosa/lips with extension to buccal mucosa	1.7429	0.3214–9.4502	0.5195

The analysis of the compromised levels revealed that level I was positive in 23.33 percent (7/30), level II in 53.3 percent (16/30), and level III in 23.33 percent (7/30). Five patients were submitted to bilateral dissections of levels I, II, III because the tumor had already crossed the facial midline.

Of the events analyzed, muscular infiltration was present in 49.04 percent of the cases, desmoplasia in 7.64 percent, vascular emboli in 12.73 percent, perineural infiltration in 27.38 percent, tumor thickness (≥4 mm) in 66.87 percent, compromised margins in 20.38 percent, pT1 in 17.19 percent, pT2 in 34.39 percent, pT3 in 40.76 percent, and pT4a in 7.64 percent of the cases. All pT4b cases were among the excluded ones.

Of these variables, desmoplasia, perineural infiltration, tumor thickness (≥4 mm), and pT4 presented with a positive association with occult cervical metastasis, with statistical significance (*p* < 0.05) (Table 3).

**Table 3:** The incidence of the studied factors in both groups, odds ratio, confidence interval and *p*-value.

*n* = 157	Group A (127)	Group B (30)	OR	95% CI	*p*-Value
Muscular infiltration	60	17	1.4603	0.6550–3.2556	0.3547
Desmoplasia	7	5	3.4286	1.0063–11.6818	0.0488
Vascular emboli	13	7	2.6689	0.9602–7.4182	0.0598
Perineural infiltration	30	13	2.4725	1.0780–5.6712	0.0326
Tumor thickness^a^	80	25	2.9375	1.0534–8.1915	0.0395
Compromised margins	27	5	0.7407	0.2592–2.1170	0.5754
pT1	25	2	0.2914	0.0650–1.3058	1.611
pT2	48	6	0.4115	0.1569–1.0787	1.806
pT3	47	17	2.2259	0.9932–4.9885	0.0520
pT4a	07	5	3.4286	1.0063–11.6818	0.0488

a ≥4 mm.

## Discussion

This case–control study was composed of 157 patients with oral squamous cell carcinoma who were initially diagnosed as N0. The sample was gathered from 1995 to 2014.

Our primary concern was to try to determine any predictive factors in histopathological reports that had a positive relationship with the occurrence of occult cervical metastasis.

The estimated rate of occult lymph node metastasis is around 15–34 percent, according to some studies.^16^, ^17^, ^18^ In our study, of the 157 patients, we have found 30 to have occult cervical metastasis after surgery, which corresponds to 19.10 percent.

It was a comprehensive study with a significant number of cases of oral cancer and with multivariate analysis.

Most tumors were located at the tongue and floor of the mouth, and according to literature these subsites are more related to a higher risk of occult cervical metastasis; however, our analysis, shown in Table 2, does not allow the clear indication of a particular oral cavity subsite as a predictive factor with statistical significance.

Regarding the factors evaluated, muscular infiltration was present in 60 cases in group A, against 17 cases in group B. The calculated odds ratio (1.4603 – CI: 0.6550–3.2556) does not allow to determine it as a risk factor for occult neck metastasis (*p* > 0.005). As a matter of fact, muscular infiltration remains an unclear factor, with some authors discussing an increased risk when the invasion exceeds 4 mm.^2^, ^11^, ^19^, ^20^

Desmoplasia and vascular emboli were related with the presence of cervical metastasis by some authors. In this study, desmoplasia occurred in seven patients in group A and five in group B, with an odds ratio of 3.4286 (CI: 1.0063–11.6818) being a risk factor for occult cervical metastasis with statistical significance (*p* = 0.0488).^10^, ^11^

Brandwein-Gensler et al. did not find statistical significance with vascular invasion. We have found 13 cases in group A and seven cases in group B with an odds ratio of 2.6689 (CI: 0.9602–7.4182), but a *p*-value of 0.0598 – not statistically significant, but with a likely tendency to it.^21^

Regarding perineural infiltration, several studies have shown it as an independent risk factor for the occurrence of occult cervical metastasis.^21^, ^22^, ^23^ We have found 30 cases in group A and 13 at group B. Odds ratio was 2.4725 (CI: 1.0780–5.6712), with a *p*-value of 0.0326 – therefore, with statistical significance and in concordance with previous studies.

Another well-studied factor is tumor thickness. It is not well defined what should be the correct parameter to consider. Different measures can be found in the literature, some with a cut-off of 3 mm, others, 4 mm.^23^, ^24^, ^25^

We adopted the 4 mm parameter since it is the most used in the current literature. We have observed 80 cases in group A and 25 in group B. The calculated odds ratio was 2.9375 (CI: 1.0534–8.1915), with a *p*-value of 0.0395, and that result seems to be rather intuitive to explain its relationship with occult cervical metastasis.

Tumor length was also analyzed based on the pTNM scale, and pT4a presented as a risk factor, with an odds ratio of 3.4286 (CI: 1.0063–11.6818) and a *p*-value of 0.0488. The other stages did not demonstrate a positive association with occult neck metastases; however, it is worth mentioning that pT3 with an odds ratio of 2.2259 (CI: 0.9932–4.9885), *p*-value 0.0520, showed a possible tendency to it as well as vascular invasion, as previously mentioned.

At last, we have analyzed if margins were compromised. We have found 27 cases in group A and five patients in group B. The estimated odds ratio was 0.7407 (CI: 0.2592–2.1170), and a *p*-value of 0.5754.

The constant evolution of the treatment of oral squamous cell carcinoma has made possible to better select patients who will undergo elective neck dissections. When correctly indicated, surgical treatment offers great outcome in five-year survival. However, this operation evolves possible major complications, such as spinal nerve lesion and significant esthetic compromise.

The use of the sentinel lymph node mapping is one technique that is being studied in head and neck cancers for a long time. It has proven its value in melanomas and breast cancers, and several centers around the globe are using it with satisfactory results.^26^, ^27^ In our service, none of the cases was submitted to this technique.

Elective neck dissection evolved levels I, II and III. This type of neck treatment is the standard procedure in our service, with its effectiveness proven in the literature.^28^, ^29^, ^30^ We found that levels I and III were equally compromised with 23.33 percent of the cases. Level II was positive in 53.3 percent of the cases. These cases were posteriorly referred to therapeutic neck treatment with modified radical neck dissection or radiochemotherapy in the cases where patients refused to undergo another surgery or did not present themselves with clinical conditions for a secondary surgery (5/30).

The present study focused only at the histopathological analysis to determine possible predictive factors for occult neck metastasis; therefore, some information, such as alcohol and tobacco consumption, was not evaluated, and neither was overall survival rate. We consider that future prospective studies correlating all of these variables are extremely necessary, considering that the vast majority of studies in this field are retrospective ones.

On the other hand, the accurate definition of predictive factors for occult cervical metastasis may also guide the selection of patients who should be referred to radiotherapy, avoiding the unnecessary exposure of low-risk patients to radiation and allowing a better regional control of the disease in those of moderate or high risk.

## Conclusion

In this study, we have found that desmoplasia, perineural infiltration, pT4a stage and tumor thickness (≥4 mm) are predictive factors for occult neck metastasis.

## Conflicts of interest

The authors declare no conflicts of interest.
